# Cannabidiol Decreases Intestinal Inflammation in the Ovariectomized Murine Model of Postmenopause

**DOI:** 10.3390/biomedicines11010074

**Published:** 2022-12-28

**Authors:** Karen Mae A. Bacalia, Kevin M. Tveter, Hayley Palmer, Jeffrey Douyere, Savannah Martinez, Ke Sui, Diana E. Roopchand

**Affiliations:** 1 Department of Food Science, NJ Institute of Food Nutrition and Health New Brunswick, Rutgers University, New Brunswick, NJ 08901, USA; 2 Graduate Program, Department of Nutritional Sciences, Rutgers University, New Brunswick, NJ 08901, USA

**Keywords:** cannabidiol, inflammation, bile acids, transcriptomics, estrogen deficiency, ovariectomized mice, postmenopause, gut organoids

## Abstract

Cannabidiol (CBD) (25 mg/kg peroral) treatment was shown to improve metabolic outcomes in ovariectomized (OVX) mice deficient in 17β-estradiol (E2). Herein, CBD effects on intestinal and hepatic bile acids (BAs) and inflammation were investigated. Following RNA sequencing of colon tissues from vehicle (VEH)- or CBD-treated sham surgery (SS) or OVX mice (*n* = 4 per group), differentially expressed genes (DEGs) were sorted in ShinyGO. Inflammatory response and bile secretion pathways were further analyzed. Colon content and hepatic BAs were quantified by LC-MS (*n* = 8–10 samples/group). Gut organoids were treated with CBD (100, 250, 500 µM) with or without TNFα and lipopolysaccharide (LPS) followed by mRNA extraction and qPCR to assess CBD-induced changes to inflammatory markers. The expression of 78 out of 114 inflammatory response pathway genes were reduced in CBD-treated OVX mice relative to vehicle (VEH)-treated OVX mice. In contrast, 63 of 111 inflammatory response pathway genes were increased in CBD-treated sham surgery (SS) mice compared to VEH-treated SS group and 71 of 121 genes were increased due to ovariectomy. CBD did not alter BA profiles in colon content or liver. CBD repressed *Tnf* and *Nos2* expression in intestinal organoids in a dose-dependent manner. In conclusion, CBD suppressed colonic inflammatory gene expression in E2-deficient mice but was pro-inflammatory in E2-sufficient mice suggesting CBD activity in the intestine is E2-dependent.

## 1. Introduction

In the United States, women typically enter perimenopause in their mid-to-late forties and reach menopause at the average age of 51 [1]. Decline in ovarian 17β-estradiol (E2) levels during the menopause transition results in uncomfortable vasomotor symptoms (e.g., hot flushes) that affect up to 80% of women and can persist for 5–13 years [2,3,4,5,6,7]. In humans and rodents, the loss of E2 has also been associated with cardiometabolic disease and bone loss [5,6,8] due to reduced gut barrier integrity leading to chronic low-grade inflammation [5,6,9,10]. During the menopause transition, hormone replacement therapy (HRT) is typically prescribed to relieve vasomotor symptoms and HRT was initially believed to mitigate chronic disease [11]. After several clinical studies, HRT appears to be most useful for management of vasomotor symptoms in women under 60 with reported benefits for the cognitive function and decreased risk of bone fracture from osteoporosis [12]. HRT dose and treatment regimen should be individualized according to medical history [13] and the lowest effective dose is generally recommended for no more than 5 years to reduce chronic disease risk [12]. HRT use in women over 60 is associated with increased risk of cancer, heart disease, and stroke [12] leaving this population with few options for symptom management and chronic disease prevention.

Cannabidiol (CBD) is a non-psychotropic phytocannabinoid derived from the industrial hemp plant (*Cannabis sativa* L.). In preclinical studies, CBD was shown to reduce inflammation [14,15,16], improve gut barrier integrity [17], and protect against bone loss [18,19,20]. Currently, CBD (i.e., Epidiolex^®^) is a FDA-approved for treatment of epilepsy-related disorders in both children and adults [21,22,23,24]. Although there are currently no studies for the effectiveness of CBD for menopausal or postmenopausal symptoms, a recent survey of 258 perimenopausal and postmenopausal women reported that 86% used medical cannabis and 79% endorsed its use to relieve menopause symptoms [25]. The effects of perorally administered CBD (25 mg/kg/day, 5 days/week for 18 weeks) or vehicle (VEH) treatment (i.e., sesame oil and peanut powder) in the ovariectomized (OVX) mouse model of postmenopause along with sham surgery (SS) controls were recently investigated [26]. Compared to VEH-treated OVX mice, the CBD-treated OVX group had improved oral glucose tolerance, increased energy expenditure, improved bone phenotypes, and the decreased markers of inflammation in bone and intestinal tissues [26]. CBD-treated OVX and SS mice had altered gut microbial communities and BA profiles [26]. Notably, the CBD-treated OVX group, but not the CBD-treated SS group, developed a bloom in *Lactobacillus* species [26]. Studies suggest that probiotic *Lactobacilli* bacteria improve gut barrier integrity leading to the lower levels of inflammatory cytokines in the gut, circulation, and bone [27,28,29,30,31]. A recent meta-analysis of randomized controlled clinical studies cautiously concluded that supplementation with probiotics could increase lumbar bone mineral density in postmenopausal women [32]. BAs modulate gut bacteria [33], inflammation [34], glucose metabolism [35], and bone turnover [36]. Compared to SS groups, several ileal BAs were increased in the VEH-treated OVX group while in CBD-treated OVX mice these BAs were normalized to levels comparable to the SS groups [26].

Oral CBD bioavailability is 6% and increases 4-fold if consumed with fat [37]. Since most ingested CBD would be in the intestinal tract, CBD may improve metabolic health by directly modulating the gut microbiota and/or decreasing intestinal inflammation. In this present study, samples collected from a previously described murine study [26] was used to investigate the response of the colonic transcriptome to CBD or VEH treatment in OVX and SS mice. Targeted metabolomics was performed in order to profile BAs in colon content and liver tissue. Finally, the direct effect of CBD on intestinal inflammation was tested in ileal gut organoids.

## 2. Materials and Methods

### 2.1. Chemicals and Materials

Experiments were performed using Optima LC-MS grade formic acid, methanol, acetonitrile, and water purchased from Fisher Scientific (Hampton, NH, USA). Oasis Prime HLB 1cc Cartridges (30 mg) were purchased from Waters (Milford, MA, USA) for filtration of phospholipids from liver samples. Corning Costar Spin-X centrifuge tubes with 0.22 μm nylon membrane (Corning, NY, USA) were used to filter BA extracts prior to injecting. CBD isolate was purchased from Bluebird Botanicals (Louisville, CO, USA). Twelve unconjugated, 9 taurine conjugated, and 6 glycine conjugated BAs were ordered from Sigma-Aldrich Inc. (St. Louis, MO, USA), Cayman Chemical (Ann Arbor, MI, USA), or Steraloids Inc. (Newport, RI, USA). Deuterated internal standards of BAs were purchased from Cayman Chemical. BA and CBD information are detailed in Table S1.

### 2.2. Animals and Treatment

Animal study protocols were approved by Rutgers institutional animal care and use committee under protocol# PROTO201900041. The details of the mouse study were previously described [26]. Briefly, at age 12 weeks, female wild-type C57BL/6J mice were ovariectomized (OVX) or underwent sham-surgery (SS). After recovery, at age 14 weeks, OVX and SS mice were subdivided to receive either CBD isolate or vehicle (VEH) treatment (*n* = 10 mice/group) for 18 weeks (5 days per week excluding weekends). A calculated volume of VEH (sesame oil) or CBD (25 mg/kg, dissolved in sesame oil, 10 mg/mL) was mixed with 100 mg of powdered peanut butter to prepare individual peroral doses, which were consumed within 1 min of offering. Mice were euthanized by CO_2_ asphyxiation and liver, and intestinal tissues were collected as previously described [26]. Individual colon segments were flushed with ice-cold, sterile PBS (pH 7.4) to collect luminal contents. Samples were placed in cryogenic tubes, snap frozen in liquid nitrogen, and stored at −80 °C until analysis.

### 2.3. Ribonucleic Acid Sequencing (RNA-Seq)

Total ribonucleic acid (RNA) from colon tissue (*n* = 4 mice/group) was extracted using RNeasy Plus Universal Mini Kit (Catalog#73404, QIAGEN, Germantown, MD, USA). The concentration, quality, and integrity of total RNA was determined using a Nano Drop 2000 spectrophotometer (Thermo Scientific, Wilmington, DE, USA) and RNA samples were provided to Azenta (South Plainfield, NJ, USA) for Standard RNA-Seq processing. Paired-end sequencing was performed using Illumina HiSeq 2 × 150 bp paired-end configuration yielding 124,739 Mbases and 415,802,210 reads.

### 2.4. RNA Sequencing Data Analysis

Raw reads were pre-processed using FastQC 0.11.9 and Java 14.0.1 software. Phred quality scores of individual sequences were checked before merging paired-end sequences with 20 bp overlap. Trimmomatic-0.39 data analysis software was used to trim adapters of paired-end sequences and assess sequence quality. HISAT2 2.1.0 was used to map reads to a reference mouse genome (grcm38 with Ensembl annotation v38.102) [38]. Differentially expressed genes (DEGs) were analyzed using DESeq2 in R (R Studio v4.2.0) [39]. Gene ontology (GO) enrichment analysis was performed using ShinyGO v0.76.3 (South Dakota State University, Brookings, SD, USA) [40] with the application of FDR correction to generate the list of pathways affected using gene ontology biological processes (GOBP) and Kyoto Encyclopedia of Genes and Genomes (KEGG) databases. The FDR q-value cut-off criteria were assigned as 0.05. The inflammatory response pathway genes were identified in the GOBP network while the bile secretion pathway genes were found in the KEGG network.

PCA plots to compare similarity/difference among RNA-Seq datasets for biological replicates within group and between groups were generated using Metaboanalyst 5.0 [41]. The fold change of DEGs was converted to logCPM (Z-score) values and heat maps of DEGs for each pathway were generated using Euclidean clustering between samples and genes using Origin Pro 2023 software (Origin Lab Corp., Northampton, MA, USA). Venn diagrams were also generated using Origin Pro.

### 2.5. LC-MS Analysis of Bile Acids

#### 2.5.1. Preparation of Liver and Colon Samples

BAs were extracted from individual liver samples (n = 9–10/group). Frozen liver tissue was sectioned on dry ice and 50–60 mg of median lobe was transferred into 2 mL bead beating tubes with 4 stainless steel beads (2.8 mm, GBSS 089-5000-11, OPS Diagnostics, Lebanon, NJ, USA) and 300 μL of water. Samples were homogenized using a 1600 MiniG^®^ (SPEX SamplePrep, Metuchen, NJ, USA) for 4 min. Homogenized tissue was transferred to microcentrifuge tubes with 300 μL of pre-dried deuterated internal standards (TCA-d4, DCA-d4, CDCA-d4, and GCDCA-d4 at 1 µg/mL each). Protein was precipitated with 99.9% acetonitrile and 1% formic acid (800 μL) and vortexed for 30 s and then placed on an orbital shaker for 1 h at 4 °C. Samples were centrifuged at 13,000× *g* for 10 min at 4 °C and supernatant was transferred to glass scintillation vials. Pellets remaining after centrifugation was resuspended in 1 mL 80% methanol and sonicated for 1 min with a Qsonica sonicator Q700 with chiller fitted with cuphorn and 8-tube holder (Cole-Palmer, Vernon Hills, IL, USA); 55% AMP, 30 s on, 59 s off, then a final 30 s on). Sonicated samples were centrifuged at 16,000× *g* for 20 min at 4 °C and supernatants were pooled and dried under speed vacuum at room temperature overnight. Samples were resuspended in 300 μL 50% methanol, placed on orbital shaker for 30 min, vortexed for 2 min, and filtered through Corning^®^ Costar^®^ Spin-X^®^ microcentrifuge tube filters (nylon membrane, pore size 0.22 μm, cat#CLS8169-200ea, Sigma-Aldrich, Darmstadt, Germany) for 5 min at 16,000× *g*. Samples were transferred into sampler vials (Cat# 6PSV9-1PSS Thermofisher,, Walthm, MA, USA) with 300 μL inserts (9 mm, C4010-630 Thermofisher, Walthm, MA, USA) for HPLC analysis. Concentrations (µg/mg tissue) were determined by dividing final concentrations by tissue weights used for extraction. For each liver sample, the limit of detection (LOD), the limit of quantification (LOQ), and the coefficient of variance (CV) are presented in Table S2. Recoveries ranged from 66–151% for TCA-d4, 35–76% for DCA-d4, 70–102% for GCDCA-d4, and 53–113% for CDCA-d4.

Individual colon content samples (*n* = 8–9/group) were collected into microfuge tubes by flushing the lumen of the colon with 1× PBS (pH 7.4). The colon content was freeze-dried in a FreeZone 1.0 L Benchtop lyophilizer (model# 7740020, LABCONCO, Kansas City, MO, USA) overnight to evaporate PBS and dry weight (mg) of colon content was recorded. To subtract weight contributed by salts in PBS, 3 tubes containing 1 mL of 1× PBS were freeze-dried and their mean weight was subtracted from dried colon content weights. Deuterated internal standards (TCA-d4, DCA-d4, GCDCA-d4, and CDCA-d4 at 1 µg/mL each) were resuspended in 50% methanol and 300 µL was added to pre-weighed microfuge tubes and dried in speed vacuum (CentriVap concentration system with cold trap, Model 7810014 and 7460020 Labconco, Kansas City, MO, USA), after which colon content (15–30 mg) was added. Then 600 μL of 90% acetonitrile/9.9% water/0.1% formic acid (*v*/*v*/*v*) was added to internal standards and dry colon content, vortexed for 1 min, and left on a benchtop shaker at 4 °C for 1 h. After extraction, samples were centrifuged at 15,000× *g* for 10 min and the supernatant was collected into a clean microfuge tube. For the second round of extraction, 700 μL of 50% methanol/50% water (*v*/*v*) was added to the pellet, vortexed for 2 min, and extract was placed in QSonica sonicator Q700 (with chiller fitted with cuphorn and 8-tube holder, Cole-Palmer, Vernon Hills, IL, USA) at 65% amplitude for 2 min. Samples were placed on shaker at 4 °C for 45 min and then centrifuged at 12,000× *g* for 10 min. Supernatants were transferred to microfuge tubes and extraction was repeated with 600 μL of 90% acetonitrile and 0.1% formic acid. Samples were vortexed for 30 sec and placed in QSonica sonicator at 65% amplitude for 1 min. Samples were placed on shaker at 4 °C for 45 min and then centrifuged at 12,000× *g* for 10 min. Supernatants from first and second extractions were pooled and solvent was evaporated to dryness using a speed vacuum (CentriVap concentration system with cold trap, Model 7810014 and 7460020 Labconco, Kansas City, MO, USA) and resuspended in 300 μL of 50% methanol. Samples were sonicated at 65% amplitude for 1 min and then filtered using 0.2 μm filters (Corning Costar Spin-x centrifuge tube filters, cat#CLS8169-200ea, Sigma-Aldrich, Darmstadt, Germany). Filtrates were centrifuged at 12,000× *g* for 10 min and transferred to HPLC vials (6PSV9-1PSS, Thermofisher, Waltham, MA, USA) fitted with 300 μL inserts (9 mm, C4010-630, Thermofisher, Waltham, MA, USA).

For each colon content sample, the limit of detection (LOD), the limit of quantification (LOQ), and the coefficient of variance (CV) are presented in Table S2. Recoveries ranged 94–156% for TCA-d4, 63–124% for DCA-d4, 47–131% for GCDCA-d4, and 75–175% for CDCA-d4.

#### 2.5.2. LC-MS Analysis

Data was generated using an Alliance e2695 HPLC system coupled to a 2998 Photodiode array detector and an Acquity QDa detector mass spectrometer equipped with an electrospray interphase (ESI, Waters, Milford, MA, USA), an autosampler, and a Vacuubrand pump (Essex, CT, USA). For each sample, technical duplicates (10 μL) were injected. The instrument and processing methods have been previously described [26]. A Cortecs C18+ column held at 40 °C (4.6 × 150 mm and 2.7 µm particle size, Waters, Milford, MA, USA) was used to separate analytes and held at the temperature of 40 °C. The mobile phase consisted of 0.1% formic acid in acetonitrile (A) and 0.1% formic acid in water (B). The flow rate was 1 mL/min. A linear gradient was used, specifically: 35–50% A over 30 min, a hold at 50% A for 1 min, an immediate transition to 65% A for 9 min, a gradual increase to 90% A over 2 min, and a hold at 90% A for 6 min. This was immediately followed by a washout with 90% A to 10% A for 6 min before returning to the initial 35% A at 54.1 min, which marked the end of each sample run. The column was allowed to equilibrate for 6 min in 35% A before the next injection. Pure compounds were used to produce standard curves for the quantification of BAs and CBD, as detailed in Table S1.

### 2.6. Ileal Organoid Experiments

Eight-month-old WT C57BL/6J female mice were euthanized by CO_2_ asphyxiation and ileal tissue was collected for crypt isolation according to established methods [42]. Crypts were collected in 1× PBS (pH 7.4, Growcells, Irvine, CA, USA), counted manually, and the concentration of 300 crypts per µL was calculated. Culture was centrifuged at 200× *g* for 3 min, PBS was aspirated, Cultrex was added to obtain a density of 150 crypts per 25 µL volume, and 48-well plates were seeded with 25 µL Cultrex per well. The plate was incubated in a 37 °C, 5% CO_2_ incubator (Galaxy 170, Eppendorf Co., New Brunswick, NJ, USA) for 30 min to allow the polymerization of Cultrex then 250 µL of 1× complete growth medium (CGM) [43] was added per well. CGM was replaced every 2 days. Organoids were passaged every 7 days (1:3 ratio). Mature day 4 organoids were treated with 0, 100, 250, or 500 µM CBD in the presence or absence of Tnfα (100 ng/mL; STEMCELL, Vancouver, BC, Canada) + lipopolysaccharide (LPS; 100 µg/mL; Sigma-Alrich, Darmstadt, Germany) to induce inflammation. Six wells were pooled to create one biological sample (*n* = 1) and treatments were performed in triplicate. Organoids from passages 10–11 were used, and the experiment was performed twice.

CBD (1 mg/mL) was dissolved in 100% methanol and then calculated volumes of this stock were used to obtain 100, 250, or 500 µM CBD concentrations (in 250 µL/well) as well as these same CBD concentrations in combination with lipopolysaccharide (LPS 2 mg/mL 0.9% NaCl stock; 10 µg/mL in CGM; Cat#L6143 Sigma-Alrich) and TNFα (100 µg/mL sterile ddH_2_O stock; 100 ng/mL in CGM; Cat#78069, STEMCELL). TNFα and LPS alone served as a positive control for inflammation. Samples were dried in speed vacuum (CentriVap concentration system with cold trap, Labconco, Kansas City, MO, USA) and resuspended in CGM media the day of treatment. Organoids were treated for 24 h, CGM was removed, and 500 µL Cultrex organoid harvesting solution (Cat# 3700-100-01, R&D Systems, Minneapolis, MN, USA) was added per well. Organoids (6 wells/treatment) were collected into 15 mL conical tubes precoated with 1× PBS and left to incubate on ice for 1 h to dissolve Cultrex. Samples were centrifuged at 500× *g* for 5 min at 4 °C, washed with 2 mL of 1× PBS, supernatant was removed, 800 µL of Qiazol was added, and samples were transferred to 1.7 mL microfuge tubes with two 2.8 mm stainless steel beads and frozen at −80 °C until RNA extraction. Samples were thawed on ice followed by vortexing for 30 s, then RNA was extracted using RNeasy plus universal mini kit (Catalog#73404, QIAGEN, Germantown, MD, USA).

### 2.7. MTT Analysis for Cell Viability

MTT, 3-(4,5-dimethylthiazol-2-yl)-2,5-diphenyltetrazolium bromide reagent (M6494, Thermofisher, Waltham, MA, USA) was diluted 5 mg/mL in sterile 1× PBS per manufacturer’s instructions. Concurrently, treatments were performed on an additional 48-well plate of organoids for MTT assay to assess viability. Three wells were used per treatment, including 100% DMSO as a positive control for toxicity. At 24 h post-treatment, 27.5 µL of MTT solution was added to the 250 µL of 1× CGM in each well, then placed in an incubator (37 °C, 5% CO_2_) for 2 h. After media and MTT solutions were removed, viable organoids appeared purple/black. Then 50 µL of 2% sodium dodecyl sulfate (SDS) was added to each well and the plate was returned to the incubator (37 °C, 5% CO2) for 1 h. After incubation, 150 μL of pure dimethyl sulfoxide (DMSO) was mixed into each well and incubated for 4 h or overnight to solubilize the formazan crystals. Once solubilization was complete, 200 μL from each well was transferred to a microplate and absorbance was measured in a multimode plate reader (CLARIOStar, BMG Labtech, Cary, NC, USA) at 562 nm.

#### 2.8. qPCR of Ileal Organoids and Liver Tissue

RNA was extracted from liver tissue (10–20 mg of right median lobe) as previously described [43]. RNA extracted from organoids or liver samples was quantified by nanodrop and 5 mg was used to prepare cDNA followed by RT-qPCR (QuantStudio 3, Thermo) as previously described [26].

TaqMan™ assay primers (Life Technologies, Carlsbad, CA, USA) used were: *Nos2* (Mm00440502_m1), *Tnfα* (Mm00443258_m1), *Il6* (Mm00446190_m1), and *Il1b* (Mm004342228_m1). *Hmbs* (Mm01143545_m1) was used as the house keeping gene.

### 2.9. Statistics

Data were analyzed using GraphPad Prism 8 software (GraphPad Software, Inc., La Jolla, CA, USA). The ROUT test was used to detect and remove any outliers. Normality and variance were tested before choosing parametric or non-parametric tests. To detect differences in liver qPCR and BA analysis, two-way ANOVA was used followed by Benjamini–Hochberg post hoc test with FDR adjustment, q < 0.05 was considered significant. Non-parametric BA data were analyzed by Kruskal–Wallis test followed Benjamini–Hochberg post hoc test with FDR adjustment. For organoid experiments, one-way ANOVA was performed followed by Tukey post hoc test, and the significance level was *p* < 0.05.

## 3. Results

### 3.1. CBD-Induced Inflammatory Response Pathway Changes in E2-Deficient and -Sufficient Female Mice

A whole transcriptomic RNA-Seq analysis of colon tissues (*n* = 4/group) was performed to investigate differential gene expression due to OVX surgery or CBD treatment. The PCA plot showed that samples within surgery and treatment groups clustered together (Figure S1).

Comparing VEH- and CBD-treated OVX groups, there was a total of 2585 differentially expressed genes (DEGs, q < 0.05) of which 1334 genes were upregulated and 1255 were downregulated (Table 1). A comparison of VEH- and CBD-treated SS groups revealed 14,508 DEGs (q < 0.05) where 964 genes were upregulated and 13,544 were downregulated (Table 1). There were 3162 DEGs (q < 0.05), 1552 increased and 1610 decreased, due to a loss of ovarian E2 (SS+VEH vs. OVX+VEH).

Gene ontology (GO) enrichment analysis was performed with ShinyGO for OVX+VEH vs. OVX+CBD, SS*+*VEH vs. SS+CBD, and SS+VEH vs. OVX+VEH using the gene ontology biological process (GOBP) database. For each of these comparisons, 1000 significantly altered GOBP pathways were identified after FDR correction and the top 20 pathways are shown in Figure S2. In a previous study, relative to VEH-treatment, CBD was found to reduce the expression of inflammatory mediators in the colon (*Il1b*, *Il6*, *Tnf*) and ileum (*Il1b*, *Il6*) in SS and/or OVX mice [26]. To find pathways related to inflammation the GOBP pathways were searched using the key word “inflammatory” and the inflammatory response pathway was found for OVX*+*VEH vs. OVX*+*CBD (pathway ranked 323), SS*+*VEH vs. SS*+*CBD (pathway ranked 219), and SS+VEH vs. OVX+VEH (pathway ranked 729). Inflammatory pathways were not detected when DEGs were mapped using KEGG.

**Figure 1:**
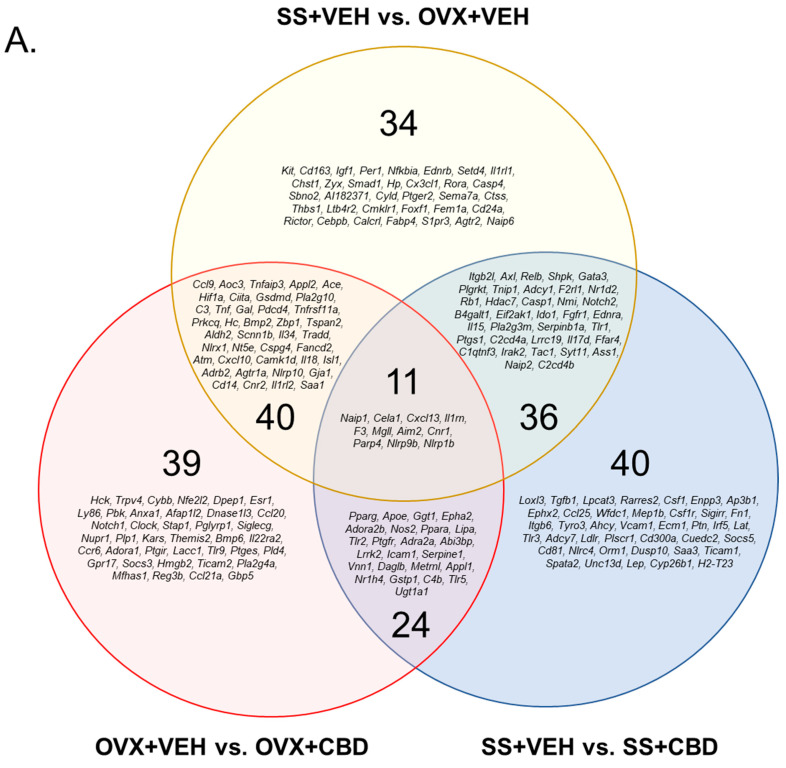

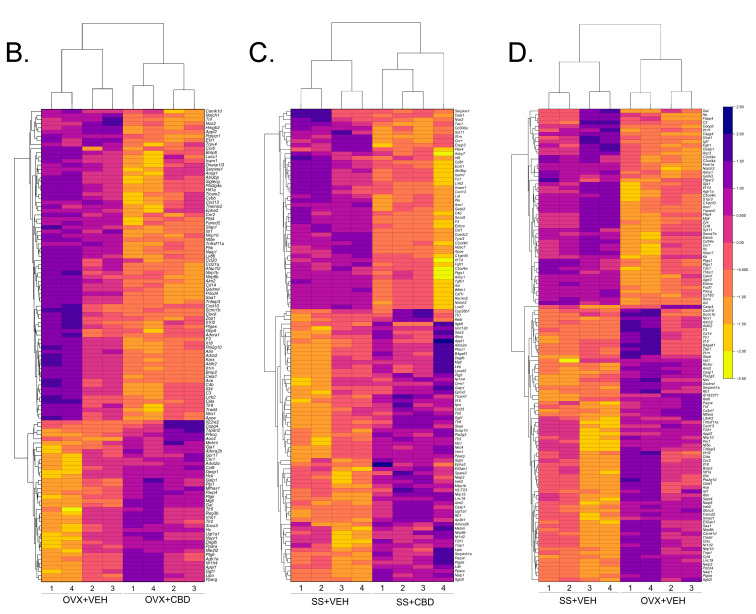
Differentially expressed inflammatory response genes. (**A**) Venn diagram showing the DEGs for each of the indicated comparisons as well as DEGs that are in common between comparisons. DEGs held in common do not necessarily indicate the same direction of change. DEGs for (**B**) OVX+VEH vs. OVX+CBD, (**C**) SS+VEH vs. SS+CBD, and (**D**) SS+VEH vs. OVX+VEH generated based on Euclidean clustering. Z-score scale indicates downregulated genes from 0 to −2.5 (orange to yellow shades) and upregulated genes from 0 to 2.5 (red to purple/navy shades).

There were 114 DEGs for the OVX+VEH vs. OVX+CBD comparison, 111 DEGs for the SS+VEH vs. SS+CBD comparison, and 121 DEGs for the SS+VEH vs. OVX+VEH comparison (Figure 1A–D). The annotations of DEGs are provided in Supplementary File S1. There were 39 DEGs uniquely altered due to the CBD treatment of OVX mice and 40 DEGs uniquely altered in the CBD-treated SS group (Figure 1A). For the OVX+VEH vs. OVX+CBD and SS+VEH vs. SS+CBD comparisons, 24 DEGs were in common and all but one (*Epha2*) were changed by CBD in the same direction indicating that the changes were independent of E2 status (Figure 1A,B and Supplementary File S1). There were 34 DEGs due to OVX alone. The remaining overlapping DEGs (40, 11, and 36) were due to either OVX or CBD treatment (Figure 1A). Consistent with prior colon tissue qPCR analysis [26], *Tnf* was significantly decreased in CBD-treated OVX mice compared to VEH-treated OVX mice (Figure 1B,D and Supplementary File S1). *Tnf* was increased in the VEH-treated OVX group compared to the VEH-treated SS group but was not detected as a DEG when comparing the VEH- and CBD-treated SS groups (Figure 1C and Supplementary File S1).

For the OVX+VEH vs. OVX+CBD comparison, CBD treatment resulted in the downregulation of 78 of the 114 differentially expressed inflammatory pathway genes (Figure 1B). In contrast, for the SS+VEH vs. SS+CBD comparison, CBD treatment resulted in the upregulation of 63 of the 111 differentially expressed inflammatory pathway genes (Figure 1C). A similar heatmap pattern was observed for the SS+VEH vs. OVX+VEH comparison where 71 of 121 differentially expressed inflammatory pathway genes were increased due to ovarian E2 deficiency (Figure 1D).

### 3.2. CBD-Induced Bile Secretion Pathway Changes in E2-Deficient and -Sufficient Female Mice

A prior study found that, compared to VEH-treated OVX mice, CBD-treated OVX mice had alterations to serum and ileal content BA profiles [26]. ShinyGO enrichment analysis followed by GOBP pathway enrichment did not uncover any hits using the keyword “bile”. Shiny GO enrichment analysis using the KEGG pathway database revealed that CBD significantly altered 134 pathways in OVX mice and 209 pathways in SS mice, while 92 pathways were altered due to ovariectomy. For each of these comparisons, the top 20 KEGG pathways were ranked based on FDR correction (Figure S3). The “Bile secretion” pathway was detected in the top 20 pathways of DEGs for OVX+VEH vs. OVX+CBD and SS+VEH vs. SS+CBD but not for SS+VEH vs. OVX+VEH (Figure S3).

**Figure 2:**
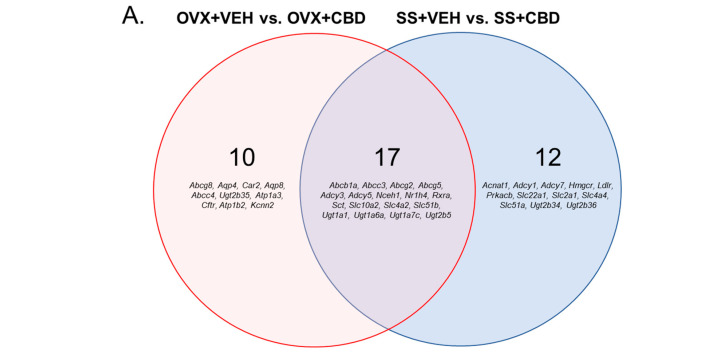

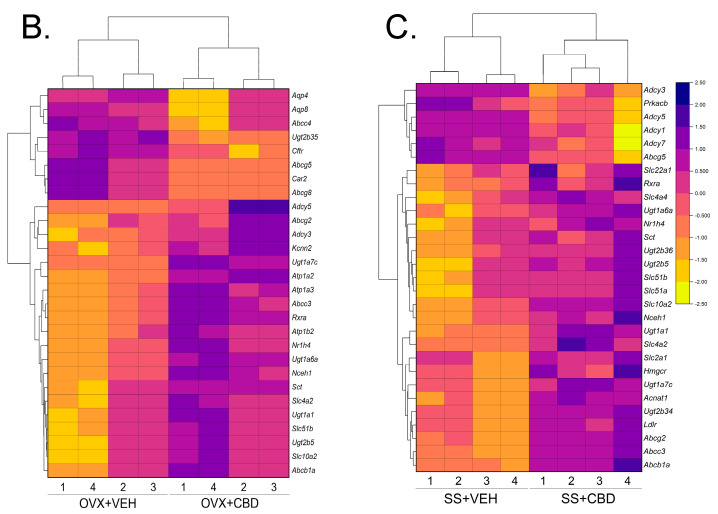
Differentially expressed bile secretion genes. (**A**) Venn diagram showing DEGs for indicated comparisons as well as DEGs that are in common between comparisons. DEGs held in common do not necessarily indicate the same direction of change. DEGs for (**B**) OVX+VEH vs. OVX+CBD and (**C**) SS+VEH vs. SS+CBD were generated based on Euclidean clustering. The Z-score scale indicates downregulated genes from 0 to −2.5 (orange to yellow shades) and upregulated genes from 0 to 2.5 (red to purple/navy shades).

Relative to VEH-treatment, the CBD-treated OVX group had 27 DEGs involved in bile secretion and the CBD-treated SS group had 29 DEGs (Figure 2A and Supplementary File S2). The OVX+VEH vs. OVX+CBD and SS+VEH vs. SS+CBD comparisons had 17 DEGs (*Abcb1a, Abcc3, Abcg2, Abcg5, Adcy3, Adcy5, Nceh1, Nr1h4, Rxra, Sct, Slc10a2, Slc4a2, Slc51b, Ugt1a1, Ugt1a6a, Ugta7c, Ugt2b5*) in common, indicating that these changes to the bile secretion pathway were CBD-induced and unrelated to surgery (Figure 2A and Supplementary File S2). For OVX+VEH vs. OVX+CBD, all 17 DEGs were upregulated due to CBD (Figure 2A,B and Supplementary File S2). For the SS+VEH vs. SS+CBD comparison, 14 DEGs were upregulated and 3 were downregulated by CBD (Figure 2A,C and Supplementary File S2).

### 3.3. Colon Content and Hepatic BA Profiles

Due to the upregulation of bile secretion pathway genes in the colon tissue, BAs in colon content were quantified. Hepatic BAs were profiled to investigate potential effects of CBD on hepatic BA production. The hepatic markers of inflammation were also investigated. The concentrations of total BAs (TBAs), primary BAs (PBAs), secondary BAs (SBAs), and conjugated BAs were similar between groups regardless of surgery or CBD treatment (Table S3). CBD did not alter the concentrations of individual BAs in colon content and liver tissue (Table S3). CBD did not induce differences in the hepatic expression of *Tnf*, *Nos2*, *Il1b*, or *Il6* (Figure S4). Compared to SS+VEH group, the OVX+VEH group showed less a hepatic expression of *Il6* (Figure S4).

### 3.4. CBD Suppressed Inflammation in Ileal Organoids

**Figure 3:**
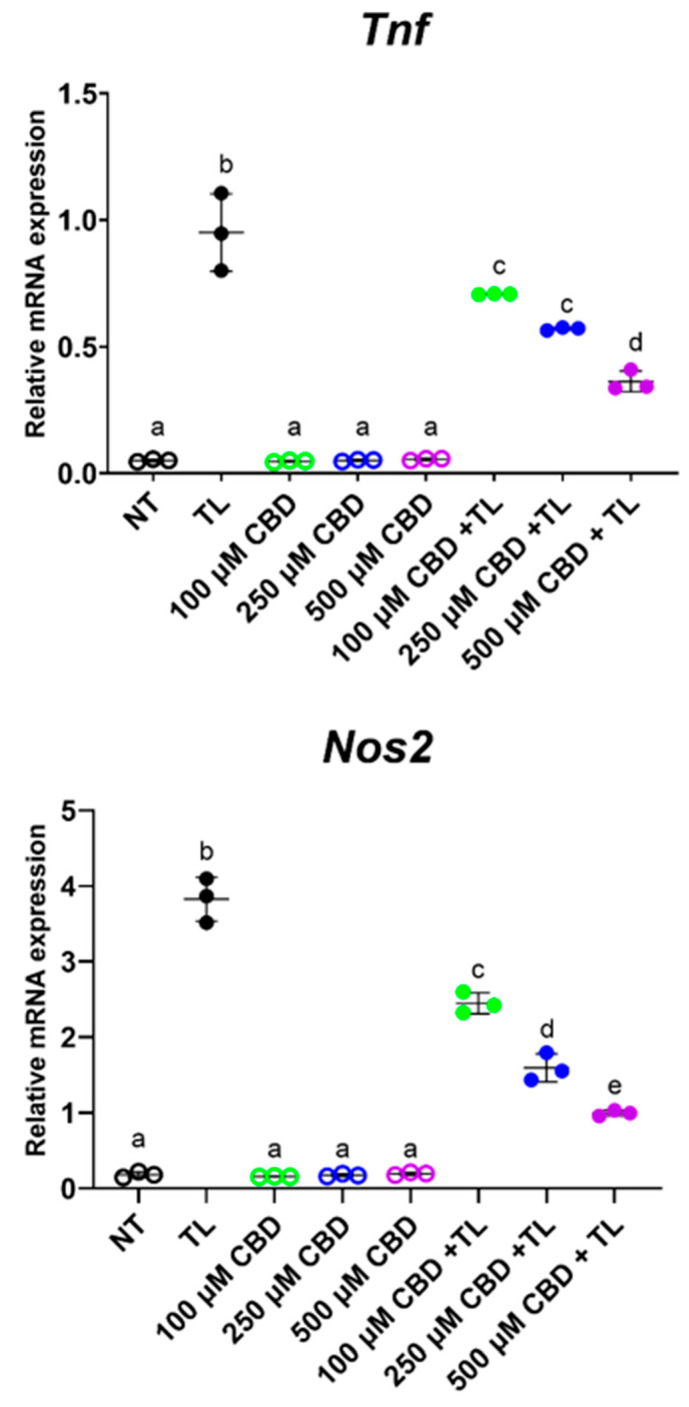
CBD decreased expression of inflammatory markers in intestinal organoids. Mature ileal organoids were treated with Tnfα + LPS (TL) to induce inflammation or treated with increasing concentrations of CBD in the absence or presence of TL. Negative controls consisted of no treatment (NT). On day 4, after organoids were passaged, organoids were incubated with treatments (*n* = 6 wells per treatment) for 24 h; each well contained approximately 100 mature organoids. Organoids were harvested and RNA was extracted for qPCR and the relative expression of target genes were determined using the 2e-Δct method. Outliers were not detected after ROUT test. Significant differences between treatments were detected using one-way ANOVA followed by a Tukey post hoc test. Different letters indicated significant difference between treatments.

Compared to vehicle treatment, the combined TNF*a* and LPS (TL) treatment of ileal organoids induced the gene expression of inflammatory markers *Nos2* and *Tnf* (Figure 3). Organoids treated with CBD concentrations of 100 or 250 µM suppressed the TL-induced expression of *Tnf* and *Nos2* where the latter showed a dose-dependent effect. Organoids treated with 500 µM CBD also appeared to decrease the TL-induced expression of *Tnf* and *Nos2*; however, this reduction may be due to the lower viability of the organoids with the 500 µM CBD dose (Figure S5). The other treatments resulted in organoid viability which was similar to NT (Figure S5). CBD treatments alone did not alter the expression of *Nos2* and *Tnf* (Figure 3). The mRNA levels of Il1b and Il6 were also assessed by qPCR but were not detected.

## 4. Discussion

The anti-inflammatory effects of CBD isolate or CBD-rich extracts have been reported [44], but the differential effects of CBD in female pre- and postmenopausal states remains largely unexplored. The decline in ovarian E2 during perimenopause and after menopause is associated with a pro-inflammatory state which promotes several metabolic disorders, including diabetes, osteoporosis, and neurodegeneration [45]. Due to the drawbacks of HRT use, especially in older postmenopausal women [12], other strategies are needed to address chronic disease burden. CBD products are currently marketed for a variety of indications, including female menopause, but often without adequate evidence [46,47]. Building upon prior work that suggested CBD may have therapeutic application in E2-deficient females [26], in this study, the RNA-Seq analysis of colon tissues revealed that the effect of CBD on inflammatory response pathways depends on E2 status. While CBD decreased the expression of inflammatory response pathway genes in E2-deficient OVX mice (Figure 1B), CBD had the opposite effect in E2-sufficient SS mice (Figure 1C), where the expression of inflammatory response genes was increased. Indeed, the CBD-treated SS and VEH-treated OVX groups showed a similar increase in inflammatory response genes (Figure 1B,D). While the loss of ovarian E2 is known to increase the expression of inflammatory markers in murine tissues [48], the elevated expression of inflammatory response pathway genes in the CBD-treated SS group was unexpected, especially given that the prior qPCR analysis of these tissues showed decreased mRNA levels of selected inflammatory markers (*Il1b*, *Il6*, and *Tnf*) in both CBD-treated OVX and SS groups relative to VEH-treated controls [26]. Notably, compared to VEH-treatment, CBD-treated SS mice had decreased mRNA levels of *Ocln* and *Tjp1*, while the latter was increased in CBD-treated OVX mice, which suggested CBD compromised gut barrier integrity in the E2-sufficient state but was beneficial in E2-deficiency [26]. It remains to be determined whether the induction of the inflammatory response pathway in CBD-treated SS mice is unique to colon tissue or whether this extends to other segments of the intestine or other tissues.

The loss of E2-producing ovary cells with increasing age in perimenopause through postmenopause leads to elevated oxidative stress, which induces inflammation [49]. In young reproductive adult females, ovarian cells have abundant mitochondria that require the high amounts of oxygen for oxidative phosphorylation and optimal cell survival [49]. Reactive oxygen species (ROS), such as superoxide ion and hydrogen peroxide, are created by oxidative phosphorylation and quenched by endogenous glutathione and dietary antioxidants (e.g., vitamins E, C, polyphenols) [49,50,51]. As aging progresses, ovarian cell membranes and mitochondria are in danger of oxidative damage due to the imperfect detoxification of oxy-radicals and reduced mitochondrial regeneration [49]. Oxidative damage leads to E2 deficiency and subsequent decline in the function and homeostasis of E2-dependent cells throughout the body [49]. CBD reduced ROS production and had a protective effect on Caco-2 monolayer integrity [17]. Together ROS and E2 deficiency may induce systemic inflammation and contribute to menopausal symptoms, such as hot flashes, an increased risk of arteriosclerosis, and decreased gut barrier integrity [17,49].

CBD is a potent antioxidant [14]. Besides its use for patients with epilepsy [21,22,23,24], CBD is being investigated for treatment of other neurodegenerative diseases, such as Huntington’s disease and schizophrenia [21,50,52,53]. CBD inhibits ROS production and modifies redox balance by activating the redox-sensitive nuclear factor erythroid 2-related factor (Nrf2) in multiple cell types [54,55]. Nrf2 transactivates several antioxidant and cytoprotective genes [55,56]. CBD was shown to reduce ROS production via the inhibition of Tnfα and iNOS [49,50], which is consistent with the CBD suppression of TL-induced inflammation in ileal organoids (Figure 3). CBD is also used for pain relief as it is a cyclooxygenase (COX)-2 inhibitor that reduces glutathione-dependent prostaglandin E2 (PGE2) signaling and subsequent inflammation [49,50]. In the present study, *Ptges*, which encodes PGE2 synthase, was downregulated in the colon tissue of CBD-treated OVX mice compared to VEH-treated OVX mice (Figure 1B and Supplementary File S1).

The presence of ROS species is sensed and monitored by the hypoxia inducible factor (HIF) pathway [57]. In hypoxic conditions, HIF1α is stable and reduces the levels of oxidative phosphorylation and ROS [57]. HIF1α stabilization plays an important role in activating osteoclast activity and bone resorption [58,59,60]. E2 destabilizes HIF1α, even under hypoxic conditions while E2-deficient OVX mice have stabilized HIF1α, which leads to bone loss [58]. Consistent with these published reports, *Hif1α* was upregulated in VEH-treated OVX mice compared to VEH-treated SS mice (Figure 1D and Supplementary File S1) and exhibited an osteoporotic bone phenotype [26]. The administration of a HIF1α inhibitor was protective against bone loss in OVX mice [58]. In the present study, *Hif1α* was downregulated in CBD-treated OVX mice compared to the VEH-treated mice (Figure 1B and Supplementary File S1) and had improved bone phenotypes [26]. It remains to be determined whether CBD directly or indirectly inhibits HIF1α.

Importantly, a physiologically beneficial level of ROS is required for pathogen resistance and cell signaling [61]. The excessive suppression of ROS was reported to induce inflammation [62], which may be the case in CBD-treated SS mice (Figure 1C). The effects of CBD on ROS in OVX and SS mice remain to be investigated.

While CBD isolate was used in this study, hemp-derived (defined as having <0.3% tetrahydrocannibinol) extract preparations contain other phytocannabinoids as well as terpenes and flavonoids [63,64]. Complex extracts are thought to have superior efficacy compared to CBD isolate preparations due to the synergistic activities of the phytochemical constituents, termed the “entourage effect” [65,66,67]. Whether other phytochemicals in a CBD-rich extract would temper CBD’s stimulation of colonic inflammation in E2-sufficent females remains to be examined. Interestingly, when male C57BL6/J mice were orally administered a CBD-rich cannabis extract (CRCE) for 5 days per week for 2 weeks, they showed a higher colonic expression of pro-inflammatory markers (*Il1ß*, *Cxcl1*, and *Cxcl2*) and a decreased expression of *Muc2*, suggesting an induction of intestinal inflammation [68]. Male mice have low circulating E2 levels making them more similar to OVX female mice then SS mice; therefore, the increased expression of colonic markers of inflammation may be due to sex-based difference in profile of hormones other than E2, CBD dose, and/or the presence of other phytochemicals in the CRCE.

The endocannabinoid system functions to maintain the homeostasis of central and peripheral tissues and displays cross-talk with estrogen signaling [69]. Endogenous cannabinoids (i.e., endocannabinoids) arachidonoylethanolamide (anandamide) and 2-arachidonoylglycerol (2-AG) are lipid messengers that signal to CB1 and CB2 endocannabinoid receptors present in central and peripheral tissues [70,71,72]. CBD activity at endocannabinoid receptors is limited but it can interact with over 65 molecular targets throughout the body [73,74]. The molecular basis of CBD bioactivity in E2-deficient vs. -sufficient states remains to be investigated.

CBD is a partial agonist for CB2, which is mainly expressed in immune cells and peripheral tissues [75]. CB2 is expressed in osteoblasts, osteoclasts, and osteocytes and is an important target for improving bone phenotypes [26,76]. In a previous study, CBD-treated OVX mice had increased femoral mRNA expression of *Cnr2*, which encodes CB2, compared to VEH-treated OVX mice and was associated with decreased bone loss [26]. In the present study, *Cnr2* was upregulated in VEH-treated OVX mice compared to the SS group and CBD treatment lead to a downregulation in the OVX mice (Figure 2B and Supplementary File S1). CBD activity at endocannabinoid receptors is limited, but it has been reported to interact with over 65 molecular targets throughout the body [73,74]. The molecular basis of CBD bioactivity in E2-deficient vs. -sufficient states remains to be investigated.

When *Corynebacterium parvum*-primed and unprimed male mice were treated with CB2 agonist WIN 55212-2 or with CB2 antagonist SR141716A, the levels of pro-inflammatory cytokines in serum was suppressed [77], suggesting that opposing effects on the CB2 receptor can result in the same outcome. Similarly, compared to VEH-treated OVX mice, CBD-treated OVX mice had increased *Cnr2* expression in bone [26] but decreased *Cnr2* expression in the colon (Figure 2B) and in both cases resulted in the reduced expression of inflammatory markers in bone and colon. Further study is needed to explain the mechanism behind these observations.

BAs are synthesized in the liver, stored in the gallbladder, and secreted into the duodenum for the digestion of lipophilic compounds [78]. 95% of Bas are reabsorbed in the ileum and return to the liver via portal circulation, while 5% enter the colon for excretion [78]. Low levels of BAs enter circulation and act as signaling molecules in diverse tissues [78]. Glycine-conjugated BAs have been correlated with increased small intestinal inflammation in rats [79]. Compared to SS groups, VEH-treated OVX mice had increased concentrations of glycine-conjugated Bas, which were reduced in CBD-treated OVX mice [26]. CBD did not alter the BA profiles in colon content (Table S3) but induced the expression of the bile secretion pathway genes in colon tissue independently of E2 status (Figure 2B,C)**.** The CBD-induced increase in bile secretion genes is likely due to it being a lipophilic compound that requires bile-mediated micelle formation for intestinal absorption [80]. An increased BA pool in the liver would be an indicator of hepatic inflammation and damage; however, CBD did not alter hepatic BA profiles in SS or OVX mice (Table S3). OVX has been associated with hepatic tissue inflammation as mice age, becoming apparent 6–7 months after the surgery [48]. There was no observation of OVX-associated increase in the expression of hepatic inflammatory markers (Figure S4), perhaps due to the mice being less than 6 months post-OVX when tissue qPCR analysis was performed.

## 5. Conclusions

In conclusion, the RNA-Seq analysis of colon tissues allowed a comprehensive investigation of CBD- and OVX-induced transcriptome changes. CBD had a potent anti-inflammatory effect in colon tissues of E2-deficient OVX mice but may contribute to inflammation in intact, E2-sufficient females. To better delineate the CBD mechanisms of action in E2-deficient and -sufficient states, additional experiments are needed to follow up on the extensive gene expression changes. The gut organoid data suggest that CBD may have a direct anti-inflammatory effect on the intestinal epithelium. Future gut organoid studies that test CBD in the absence and presence of E2 treatment would contribute to understanding CBD actions in E2-deficient and -sufficient states. In both the SS and OVX groups, CBD induced similar changes to genes related to bile secretion, indicating changes that were independent of E2 status. OVX or CBD treatment did not alter BA levels in liver or colon content, suggesting that previously observed CBD-induced changes in ileal and serum BAs [26] are more relevant. There is currently widespread use but inadequate investigation of CBD and CBD-rich extracts/products in the menopause and postmenopause [25]. Whether CBD and/or other phytocannabinoids have differential effects in women based on E2 status warrants further study. Given that HRT is not recommended for the prevention of chronic conditions in postmenopausal women, CBD may offer a therapeutic option; however, more research is needed to assist women in making better-informed judgements about individualized CBD risks and benefits.

## Figures and Tables

**Table 1:** Differentially Expressed Genes in Total Transcriptome.

	Upregulated	Downregulated	Total Changed
OVX+VEH vs. OVX+CBD	1334	1255	2589
SS+VEH vs. SS+CBD	964	13,544	14,508
SS+VEH vs. OVX+VEH	1552	1610	3162

The number of differentially expressed genes (DEGs) generated from EdgeR analysis of total transcriptome based on surgery or treatment comparison. Significance based on false discovery rate (FDR) correction (q < 0.05). OVX: ovariectomized; SS: sham surgery; VEH: vehicle treatment; CBD: cannabidiol treatment.

## Data Availability

The accession number for deposited RNA sequences is PRJNA908843 and can be found at https://www.ncbi.nlm.nih.gov/bioproject/PRJNA908843. The data can be accessed on 1 January 2023.

## Supplementary Materials

The following supporting information can be downloaded at: https://www.mdpi.com/article/10.3390/biomedicines11010074/s1, Figure S1: Two-dimensional PCA score plots comparing difference/similarity between collection of DEGs within and between indicated biological samples; Figure S2: Top 20 enriched GOBP pathways generated in ShinyGO; Figure S3: Top 20 enriched KEGG pathways generated in ShinyGO; Figure S4: qPCR of inflammatory markers in liver tissue; Figure S5: Cell viability of ileal organoids. Table S1: Bile acid information, cannabidiol, and HPLC processing method parameters; Table S2: Calibration curves, limit of detection and quantification, and coefficient of variance for bile acid and cannabidiol analysis; Table S3: Bile acid concentrations in colon content and liver. Supplementary File: Gene annotations of DEGs in inflammatory response (S1) and bile secretion (S2) pathways.
