# Supplementary material for: Cannabidiol Decreases Intestinal Inflammation in the Ovariectomized Murine Model of Postmenopause
Source: Biomedicines. 2022 Dec 28;11(1):74. doi: 10.3390/biomedicines11010074 (PMC9855871; doi:10.3390/biomedicines11010074)
Supplement: Supplementary file 1 [file biomedicines-11-00074-s001.zip › biomedicines-2102859-supplementary.pdf]

Table S1. Bile acid information, Cannabidiol, and HPLC processing method parameters.

No.	Bile acid common name	Abbreviation	Supplier	*m/z* [1] [M - H]	*m/z* [2] [M + H]	CV	Rt (min)	Internal standard [3]
1	Tauro-ω-muricholic acid	TωMCA	Steraloids	514.3	-	30	4.23	d4-TCA
2	Glycohyocholic acid	GHCA	Cayman	464.3	-	30	4.43	d4-GCDCA
3	Tauro-α-muricholic acid	TαMCA	Steraloids	514.3	-	30	4.60	d4-TCA
4	Tauro-β-muricholic acid	TβMCA	Steraloids	514.3	-	30	5.06	d4-TCA
5	Glycocholic acid	GCA	Sigma	464.3	-	30	5.64	d4-GCDCA
6	ω-muricholic acid	ωMCA	Steraloids	407.3	-	50	5.68	d4-TCA
7	Glycoursodeoxycholic acid	GUDCA	Steraloids	-	414.3/899.7	30	6.19	d4-GCDCA
8	α-muricholic acid	αMCA	Steraloids	407.3	-	50	6.21	d4-TCA
9	β-muricholic acid	βMCA	Steraloids	407.3	-	50	7.33	d4-TCA
**10**	**[3]Taurocholic acid-d4**	**d4-TCA**	**Cayman**	**518.4**	**-**	**15**	**8.08**	**-**
11	Taurocholic acid	TCA	Steraloids	514.3	-	30	8.22	d4-TCA
12	Tauroursodeoxycholic acid	TUDCA	Steraloids	498.3	-	30	8.54	d4-TCA
13	Taurohyodeoxycholic acid	THDCA	Steraloids	-	464.3	30	8.56	d4-TCA
14	Hyocholic acid (γ-MCA)	HCA	Cayman	-	355.3	30	8.60	d4-TCA
15	Cholic acid	CA	Sigma	407.3	-	50	9.98	d4-TCA
16	Murideoxycholic acid	MDCA	Cayman	-	356.3	30	10.94	d4-TCA
17	Glycochenodeoxycholic acid	GCDCA	Sigma	-	414.3/899.7	30	12.13	d4-GCDCA
**18**	**[3]Glycochenodeoxycholic acid-d4**	**d4-GCDCA**	**Cayman**	**-**	**436.4**	**15**	**13.2**	**-**

19	Ursodeoxycholic acid	UDCA	Steraloids	-	357.3	30	12.15	d4-CDCA
20	Hyodeoxycholic acid	HDCA	Sigma	-	357.3	30	12.79	d4-CDCA
21	Glycodeoxycholic acid	GDCA	Cayman	-	414.3/450.3	30	13.45	d4-GCDCA
22	Nutriacholic acid	NCA	Steraloids	-	355.2/373.3	30	15.80	d4-CDCA
23	Taurochenodeoxycholic acid	TCDCA	Sigma	-	464.3	30	16.06	d4-CDCA
24	Taurodeoxycholic acid	TDCA	Sigma	-	464.3	30	17.90	d4-DCA
**25**	**[3]Chenodeoxycholic acid-d4**	**d4-DCA**	**Cayman**	**-**	**361.3**	**30**	**20.92**	**-**
26	Chenodeoxycholic acid	CDCA	Sigma	-	357.3	30	21.09	d4-DCA
**27**	**[3]Deoxycholic acid-d4**	**d4-DCA**	**Steraloids**	**-**	**361.3**	**30**	**22.07**	**-**
28	Deoxycholic acid	DCA	Sigma	-	357.3	30	22.25	d4-DCA
29	Glycolithocholic acid	GLCA	Cayman	-	416.4/867.7	30	27.80	d4-GCDCA
30	Isodeoxycholic acid	isoDCA	Cayman	-	358.3	30	31.30	d4-DCA
31	Taurolithocholic acid	TLCA	Sigma	482.3	-	30	33.70	d4-DCA
32	[4]Cannabidiol	CBD	Bluebird	-	315.2	15	38.80	-

Bile acids (BA) were injected in negative and positive ionization modes. Selective ion recordings (SIRs) were optimized and used for quantification of each BA species. Base peak (s) were used for quantification of each individual BA is reported on the table. Peak area (µV*s) was identified using ApexTrek algorithm in Empower 3 software. A smoothing algorithm of 19 was applied to each SIR to resolve peaks. A total of 23 SIRs were established; each corresponding to the reported mass (m/z- or m/z+) of each BA analyzed in circulation or ileal content. The same SIR channel was used for BAs with the same ionized isotopic mass. This panel of BA species covers 27 BA species reported to be detected in murine/human serum and intestinal content.

[1]m/z [M - H] is negative ion mode, [2]m/z [M + H] is positive ion mode**,** [3]Deoxycholic acid-d4, [3]Chenodeoxycholic acid-d4, Glycochenodeoxycholic acid-d4, and [3]Taurocholic acid-d4 (IS STDs) were used as internal standards to calculate both matrix effect and percent recovery of samples during method development. The IS STDs was used to calculate response factors between samples and external standard curve with internal standard calibrants based on similar chemical structure and retention time.

[4]Cannabidiol was determined using an external standard curve with no internal standard calibrant.

Table S2. Calibration curves, limit of detection and quantification, and coefficient of variance for bile acid and cannabidiol analysis.

			Colon Content BAs					Liver BAs				
No.	Bile acid (BA) Common Name	Abbreviation	Calibration Curves [a]	R^2	LOD [c]	LOQ [d]	CV (%) [b]	Calibration Curves [a]	R^2	LOD [c]	LOQ [d]	CV (%) [b]
1	Tauro-ω-muricholic acid	TωMCA	y = 0.9945x + 0.0493<br>y = 0.9456x + 0.082	.9975<br>.9906	0.01<br>0.01	0.245<br>0.233	4.87	y = 1.5636x – 0.0833<br>y = 1.2704x + 0.0285	.994<br>.9995	0.01<br>0.01	0.248<br>0.305	34.6
2	Glycohyocholic acid	GHCA	y = 0.767x + 0.0004<br>y = 0.739x + 0.0466	1<br>.9998	0.01<br>0.01	0.424<br>0.217	2.43	y = 0.1724x – 0.0031<br>y = 0.1669x + 0.0084	.9889<br>.9979	0.01<br>0.01	0.225<br>0.442	22.2
3	Tauro-α-muricholic acid	TαMCA	y = 1.4554x + 0.0688<br>y = 1.4034x + 0.0986	.9971<br>.9931	0.01<br>0.01	0.144<br>0.150	0.86	y = 2.1439x – 0.0805<br>y = 2.0928x + 0.0933	.9928<br>.9988	0.01<br>0.01	0.042<br>0.043	32.9
4	Tauro-β-muricholic acid	TβMCA	y = 2.533x + 0.1508<br>y = 2.4369x + 0.2013	.9964<br>.9915	0.01<br>0.01	0.140<br>0.146	1.15	y = 3.1415x – 0.1131<br>y = 3.0565x + 0.1213	.9924<br>.9989	0.01<br>0.01	0.018<br>0.002	34.7
5	Glycocholic acid	GCA	y = 0.1018x - 0.0028<br>y = 0.1089x - 0.0038	.9984<br>.999	0.01<br>0.01	0.069<br>0.064	4.73	y = 0.1028x – 0.0113<br>y = 0.1x – 0.0065	.9943<br>.9956	0.01<br>0.01	0.330<br>0.339	22.3
6	ω-muricholic acid	ωMCA	y = 0.1657x + 0.0057<br>y = 0.1784x + 0.0117	.9977<br>.9911	0.01<br>0.01	0.256<br>0.237	4.60	y = 0.3366x – 0.0041<br>y = 0.3195x – 0.0038	.988<br>.9919	0.01<br>0.01	0.006<br>0.007	60.3
7	Glycoursodeoxycholic acid	GUDCA	y = 1.3879x - 0.0274<br>y = 1.4141x - 0.0281	.999<br>.999	0.001<br>0.001	0.004<br>0.004	2.24	y = 1.2783x – 0.0746<br>y = 1.2561x + 0.0428	.9948<br>.9993	0.001<br>0.001	0.176<br>0.179	14.4
8	α-muricholic acid	αMCA	y = 0.2289x + 0.0154<br>y = 0.2466x + 0.0248	.9936<br>.9842	0.01<br>0.01	0.290<br>0.269	2.21	y = 0.5326x – 0.0272<br>y = 0.5127x – 0.0104	.9931<br>.9968	0.01<br>0.01	0.223<br>0.232	48.2
9	β-muricholic acid	βMCA	y = 0.2869x + 0.003<br>y = 0.3026x + 0.0171	.9988<br>.993	0.001<br>0.001	0.165<br>0.166	2.45	y = 0.603x – 0.0232<br>y = 0.4795x – 0.0133	.9923<br>.9918	0.001<br>0.001	0.116<br>0.146	54.6
10	Taurohyodeoxycholic acid	THDCA	y = 4.2486x + 0.2168<br>y = 4.2151x + 0.3161	.9984<br>.994	0.01<br>0.01	0.165<br>0.166	1.69	y = 7.7358x – 0.2816<br>y = 7.8549x + 1.2609	.9934<br>.9664	0.01<br>0.01	0.895<br>0.882	61.4
11	Tauroursodeoxycholic acid	TUDCA	y = 1.0336x + 0.0609<br>y = 1.0526x + 0.0834	.9863<br>.9865	0.001<br>0.001	0.153<br>0.151	0.32	y = 0.053x + 0.0093<br>y = 0.0507x + 0.0223	.9504<br>.9474	0.001<br>0.001	1.73<br>1.81	17.5
12	Taurocholic acid	TCA	y = 1.0898x + 0.0114<br>y = 1.0697x + 0.0419	.9993<br>.9969	0.001<br>0.001	0.198<br>0.201	2.04	y = 1.3817x – 0.1177<br>y = 1.3671x + 0.0257	.9947<br>.9989	0.001<br>0.001	0.471<br>0.476	24.6
13	Hyocholic acid (γ-MCA)	HCA	y = 2.3533x + 0.1373<br>y = 2.2539x + 0.1857	.9967<br>.9934	0.001<br>0.001	0.145<br>0.151	4.79	y = 2.6155x – 0.0962<br>y = 3.0523x + 0.0887	.9939<br>.9981	0.001<br>0.001	0.020<br>0.017	6.22
14	Cholic acid	CA	y = 0.2664x + 0.0013<br>y = 0.27x + 0.0014	.9993<br>.9951	0.001<br>0.001	0.004<br>0.004	2.31	y = 0.4272x – 0.0226<br>y = 0.3488x + 0.0107	.9936<br>.9993	0.001<br>0.001	0.197<br>0.241	32.06
15	Taurochenodeoxycholic acid	TCDCA	y = 1.1851x - 0.0247<br>y = 1.1173x - 0.0025	.9935<br>.998	0.01<br>0.01	0.132<br>0.140	3.23	y = 0.9568x + 0.0925<br>y = 1.2281x + 0.0466	.9508<br>.999	0.01<br>0.01	0.339<br>0.264	7.85
16	Ursodeoxycholic acid	UDCA	y = 0.7141x - 0.0015<br>y = 0.7244x - 0.0005	.9996<br>.9993	0.001<br>0.001	0.009<br>0.009	1.98	y = 0.6566x + 0.0812<br>y = 0.8422x + 0.0469	.9488<br>.9984	0.001<br>0.001	0.369<br>0.288	9.82
17	Glycochenodeoxycholic acid	GCDCA	y = 1.3782x - 0.0523<br>y = 1.7036x - 0.0292	.9979<br>.9991	0.005<br>0.005	0.118<br>0.096	3.56	y = 1.3855x – 0.1369<br>y = 1.2678X + 0.0446	.9944<br>.9994	0.005<br>0.005	0.471<br>0.515	9.95
18	Hyodeoxycholic acid	HDCA	y = 1.0789x + 0.0159<br>y = 1.1085x + 0.0049	.9997<br>.9994	0.001<br>0.001	0.072<br>0.070	1.89	y = 0.8156x + 0.0851<br>y = 1.0557x + 0.0632	.9533<br>.9975	0.001<br>0.001	0.190<br>0.147	6.32
19	Glycodeoxycholic acid	GDCA	y = 1.8754x - 0.0664<br>y = 1.8796x - 0.0467	.9982<br>.9987	0.005<br>0.005	0.074<br>0.074	1.88	y = 1.4096x – 0.0551<br>y = 1.4826x + 0.0272	.9926<br>.9995	0.005<br>0.005	0.140<br>0.133	10.8
20	Nutriacholic acid	NCA	y = 0.055x + 0.0036<br>y = 0.0648x - 0.0029	.997<br>.9952	0.01<br>0.01	0.089<br>0.07	5.70	y = 0.0066x + 0.0004<br>y = 0.0085x + 0.0005	.9688<br>.9887	0.01<br>0.01	0.107<br>0.083	11.56
21	Taurodeoxycholic acid	TDCA	y = 0.7521x + 0.0241<br>y = 0.8036x - 0.0429	.9986<br>.9955	0.005<br>0.005	0.177<br>0.165	0.63	y = 1.2396x – 0.2307<br>y = 0.9841x – 0.0126	.9836<br>.9995	0.005<br>0.005	1.244<br>1.567	5.60
22	Murideoxycholic acid	MDCA	y = 0.7761x + 0.0199<br>y = 0.7653x + 0.0439	.9984<br>.9937	0.005<br>0.005	0.218<br>0.221	4.96	y = 1.0271x – 0.0481<br>y = 1.0384x + 0.1478	.9942<br>.9778	0.005<br>0.005	0.686<br>0.679	9.31

23	Isodeoxycholic acid	isoDCA	y = 3.638x - 0.2512<br>y = 9.422x + 0.3299	.9994<br>.9990	0.01<br>0.01	0.044<br>0.059	2.88	y = 1.6908x – 0.3821<br>y = 1.3324x – 0.1105	.9692<br>.9916	0.01<br>0.01	1.13<br>1.44	9.58
24	Chenodeoxycholic acid	CDCA	y = 1.3781x - 0.0563<br>y = 1.4025x - 0.0603	.9967<br>.9968	0.005<br>0.005	0.021<br>0.020	3.37	y = 1.1167x + 0.0537<br>y = 1.445x – 0.0006	.9702<br>.9993	0.005<br>0.005	0.336<br>0.260	6.70
25	Deoxycholic acid	DCA	y = 0.9074x - 0.0177<br>y = 0.9092x - 0.02	.999<br>.9985	0.001<br>0.001	0.018<br>0.018	4.55	y = 1.1816x – 0.198<br>y = 0.9355x + 0.0152	.9838<br>.9995	0.001<br>0.001	1.09<br>1.38	7.79
26	Glycolithocholic acid	GLCA	y = 2.2195x - 0.07<br>y = 2.2343x - 0.0369	.9985<br>.9993	0.01<br>0.01	0.105<br>0.104	2.67	y = 2.0833x – 0.1061<br>y = 2.0557x + 0.1212	.9943<br>.9975	0.01<br>0.01	0.051<br>0.052	7.69
27	Taurolithocholic acid	TLCA	y = 0.1414x - 0.0088<br>y = 0.2697x - 0.0028	.9997<br>.992	0.01<br>0.01	0.299<br>0.157	3.20	y = 0.6103x – 0.112<br>y = 0.7486x – 0.0267	.9868<br>.9992	0.01<br>0.01	0.988<br>0.805	59.2
28	[e] Cannabidiol	CBD	y = 2E+07x + 905591<br>y = 2E+07x + 875327	.9968<br>.9969	0.01<br>0.01	0.011<br>0.011	0.004	y = 1E+07x – 541410<br>y = 1E+07x - 416771	.9939<br>.999	0.01<br>0.01	0.088<br>0.088	1.69

An 8-point external standard curve (0.001µg/mL to 5 µg/mL) of bile acid (BA) standards was injected in duplicate at the beginning, middle, and end of the sample sets. Linear calibration curves were generated using external standards calibrated with deuterated internal standards (1 µg/mL) to calculate response factors for each BA species in colon content and liver samples. BA species are listed by common name and respective abbreviation.

[a] Peak area (µV*s) of each BA standard concentration was averaged to generate a linear calibration curve (from lowest to highest concentration; µg/mL) for each sample set. A total of four calibration curves (2 per tissue) were collectively used to quantify BA concentrations (µg/mg colon content or µg/mg of liver). R^2 values are listed next to each respective linear calibration curve.

[b] Coefficient of variance (CV) for each bile acid was determined by comparing mean and standard deviation of peak areas (µV*s) for quality control (QC) samples and BA standards.

[c] Limit of detection (LOD) for each BA species was determined by the lowest quantifiable peak discernible via manual integration with a signal to noise (S/N) ratio, greater than 2, for each single ion recording (SIR). LOD values correspond to BA standards detected in both injections for each standard curve point.

[d] Limit of quantification (LOQ) for each linear calibration curve was determined using the equation (10*σ/s), for which 'σ' is the standard deviation of the response factor for each BA standard curve, and 's' is the slope of the y-intercept for each linear calibration curve.

[e] Cannabidiol (CBD) concentrations (µg/mg tissue) was determined using a linear external standard curve of CBD isolate (Bluebird Botanicals, Louisville, CO) that was injected and prescreened for contaminate peaks/cannabinoid compounds.

Table S3. Bile acid concentrations in colon content and liver.

	Group:	SS + VEH (n=9)	SS + CBD (n=9)	OVX + VEH (n=9)	OVX + CBD (n=8)	SS + VEH (n=10)	SS + CBD (n=10)	OVX + VEH (n=9)	OVX + CBD (n=9)
**BA**	**Type**	**Colon Content BA Concentration (μg/mg colon content)**				**Liver BA Concentration (μg/mg liver)**			
UDCA	1°	0.001 ± 0.001	0.001 ± 0.0005	0.0003 ± 0.0005	0.0004 ± 0.0004	$2.87x10^{-5} \pm 1.72x10^{-5}$	$2.16x10^{-5} \pm 1.27x10^{-5}$	$2.8x10^{-5} \pm 1.5x10^{-5}$	$2.3x10^{-5} \pm 1.2x10^{-5}$
CDCA	1°	0.0004 ± 0.0005	0.001 ± 0.004	0.0002 ± 0.0003	0.0003 ± 0.0005	$7.4x10^{-6} \pm 3.5 x 10^{-6}$	$7x10^{-6} \pm 3x10^{-6}$	$8.81x10^{-6} \pm 4.29x10^{-6}$	$8.19x10^{-6} \pm 3.07x10^{-6}$
GUDCA	1°	0.003 ± 0.0004[b]	0.002 ± 0.001[b]	0.001 ± 0.001[a,b]	0.001 ± 0.0001[a]	$6.46x10^{-7} \pm 2.53x10^{-7}$	$6.73x10^{-7} \pm 1.40 x10^{-7}$	$6.04x10^{-7} \pm 1.72x10^{-7}$	$5.64x10^{-7} \pm 0.90x10^{-7}$
GCDCA	1°	0.0001 ± 0.002[b]	0.0001 ± 0.001[b]	$2x10^{-5} \pm 6x10^{-5}$[a]	$4x10^{-5} \pm 7x10^{5}$[a,b]	N.D.	N.D.	N.D.	N.D.
TUDCA	1°	0.0002 ± 0.0001	0.0002 ± $6x10^{-5}$	0.0002 ± 0.0002	0.0002 ± 0.0001	0.003 ± 0.003	0.003 ± 0.002	0.003 ± 0.002	0.003 ± 0.001
TCDCA	1°	$4 x10^{-5} \pm 3 x10^{-5}$	$1.5x10^{-5} \pm 1x10^{-5}$	$1.4x10^{-5} \pm 2x10^{-5}$	$2x10^{-5} \pm 2x10^{-5}$	0.0002 ± 0.0001	0.0001 ± 0.0001	0.0001 ± 0.0001	0.0001 ± 0.0001
αMCA	1°	0.02 ± 0.01	0.018 ± 0.01	0.02 ± 0.01	0.01 ± 0.005	$5x10^{-5} \pm 2x10^{-5}$	$3x10^{-5} \pm 2x10^{-5}$	$5x10^{-5} \pm 3x10^{-5}$	$5x10^{-5} \pm 3x10^{-5}$
βMCA	1°	0.02 ± 0.01	0.02 ± 0.01	0.01 ± 0.01	0.01 ± 0.005	0.0004 ± 0.0002	0.0002 ± 0.0001	0.0003 ± 0.0004	0.0003 ± 0.0001
CA	1°	0.011 ± 0.011	0.012 ± 0.012	0.005 ± 0.007	0.009 ± 0.013	0.0005 ± 0.0002	0.0003 ± 0.0001	0.0005 ± 0.0005	0.0004 ± 0.0002
GHCA	1°	N.D.	N.D.	N.D.	N.D.	N.D.	N.D.	N.D.	N.D.
GCA	1°	$1.4x10^{-5} \pm 7x10^{-6}$	$1.5x10^{-5} \pm 7x10^{-6}$	$6.5x10^{-6} \pm 7x10^{-6}$	$1.3x10^{-5} \pm 1x10^{-5}$	$9.85x10^{-6} \pm 4.85x10^{-6}$	$7.25x10^{-6} \pm 4.90x10^{-6}$	$5.5x10^{-6} \pm 4.0x10^{-6}$	$6.2x10^{-6} \pm 3.6x10^{-6}$
TαMCA	1°	0.0007 ± 0.001	0.0004 ± 0.0003	0.0004 ± 0.0005	0.0004 ± 0.0005	0.0002 ± 0.0002	0.00018 ± 0.00001	$16x10^{-5} \pm 8x10^{-5}$	$18x10^{-5} \pm 9x10^{-5}$
TβMCA	1°	0.001 ± 0.002	0.0005 ± 0.0002	0.0005 ± 0.0007	0.0003 ± 0.0002	0.0005 ± 0.0003	0.0004 ± 0.0003	0.0003 ± 0.0002	$2x10^{-4} \pm 1x10^{-4}$
TCA	1°	0.0005 ± 0.0003	0.0004 ± 0.0002	0.0003 ± 0.0003	0.0003 ± 0.0002	0.002 ± 0.002	0.003 ± 0.001	0.002 ± 0.001	0.002 ± 0.001
NCA	2°	0.0003 ± 0.001	0.0002 ± 0.0003	0.0001 ± 0.0001	0.0002 ± 0.0004	0.0001 ± 0.0001	$4.33x10^{-5} \pm 9.32x10^{-5}$	0.0002 ± 0.0001	$6.38x10^{-5} \pm 8.12x10^{-5}$
HCA	2°	0.0005 ± 0.0002	0.0005 ± 0.0003	0.0006 ± 0.0006	0.0005 ± 0.0003	$9.99x10^{-7} \pm 0.3x10^{-7}$	$1.26x10^{-6} \pm 3.37x10^{-6}$	$3.37x10^{-7} \pm 7.08x10^{-7}$	$9.23x10^{-7} \pm 0.16x10^{-7}$
MDCA	2°	0.0013 ± 0.0013	0.0012 ± 0.0001	0.001 ± 0.001	0.0007 ± 0.0004	$3.8x10^{-5} \pm 1.8x10^{-5}$	$2.5 x10^{-5} \pm 1.4x10^{-5}$	$3.6x10^{-5} \pm 2.4x10^{-5}$	$3.7x10^{-5} \pm 1.6x10^{-5}$
HDCA	2°	0.007 ± 0.003	0.007 ± 0.004	0.008 ± 0.005	0.009 ± 0.004	$4.9x10^{-5} \pm 2.7x10^{-5}$	$3.29x10^{-5} \pm 1.60x10^{-5}$	$4.52x10^{-5} \pm 2.18x10^{-5}$	$6x10^{-5} \pm 3x10^{-5}$
DCA	2°	0.04 ± 0.02	0.04 ± 0.03	0.03 ± 0.02	0.03 ± 0.008	$6.5x10^{-5} \pm 6.2x10^{-5}$	$4.5x10^{-5} \pm 3.0 x10^{-5}$	$4.4x10^{-6} \pm 2.7x10^{-6}$	$6.7x10^{-6} \pm 6.4x10^{-6}$
isoDCA	2°	0.005 ± 0.003	0.007 ± 0.005	0.007 ± 0.003	0.007 ± 0.002	N.D.	N.D.	N.D.	N.D.
GDCA	2°	0.0004 ± 0.0004	0.0004 ± 0.0004	0.0005 ± 0.0005	0.0005 ± 0.0002	N.D.	N.D.	N.D.	N.D.
GLCA	2°	N.D.	N.D.	N.D.	N.D.	N.D.	N.D.	N.D.	N.D.
TDCA	2°	0.0004 ± 0.0005	0.0003 ± 0.0003	0.001 ± 0.001	0.0002 ± 0.0001	0.0003 ± 0.0002	$16x10^{-5} \pm 8x10^{-5}$	0.0002 ± 0.0001	$17x10^{-5} \pm 8x10^{-5}$
TLCA	2°	N.D.	N.D.	N.D.	N.D.	N.D.	N.D.	N.D.	N.D.
ωMCA	2°	0.04 ± 0.01	0.04 ± 0.01	0.03 ± 0.01	0.03 ± 0.008	$7.39x10^{-5} \pm 3.59x10^{-5}$	$5.61x10^{-5} \pm 3.45x10^{-5}$	$6.58x10^{-5} \pm 2.74 x10^{-5}$	$7.3x10^{-5} \pm 4.3x10^{-5}$
THDCA	2°	0.002 ± 0.001	0.002 ± 0.0001	0.002 ± 0.002	0.002 ± 0.001	$6.9x10^{-5} \pm 5.7x10^{-5}$	$5.52x10^{-5} \pm 4.26x10^{-5}$	$4.07x10^{-5} \pm 1.54x10^{-5}$	$4.83x10^{-5} \pm 2.22x10^{-5}$
TωMCA	2°	0.005 ± 0.01	0.003 ± 0.003	0.001 ± 0.001	0.002 ± 0.002	0.0005 ± 0.0003	0.0004 ± 0.0003	0.0003 ± 0.0001	0.0004 ± 0.0002
**Unconjugated BAs**		0.14 ± 0.04	0.14 ± 0.06	0.11 ± 0.06	0.11 ± 0.03	0.0012 ± 0.0005	0.0008 ± 0.0004	0.001 ± 0.001	0.0010 ± 0.0004
**Conjugated BAs**		0.008 ± 0.01	0.005 ± 0.004	0.003 ± 0.004	0.003 ± 0.003	0.0076 ± 0.0005	0.007 ± 0.004	0.006 ± 0.003	0.006 ± 0.002
**Glycine-conjugated**		$5x10^{-5} \pm 4x10^{-5}$	$4x10^{-5} \pm 2x10^{-5}$	$2.3x10^{-5} \pm 1x10^{-5}$	$3x10^{-5} \pm 1x10^{-5}$	$1.05x10^{-5} \pm 4.98x10^{-5}$	$7.92x10^{-6} \pm 4.98x10^{-6}$	$6.1x10^{-5} \pm 4.1x10^{-5}$	$6.75x10^{-5} \pm 3.60x10^{-5}$
**Taurine-conjugated**		0.008 ± 0.01	0.004 ± 0.004	0.003 ± 0.004	0.003 ± 0.003	0.008 ± 0.006	0.007 ± 0.004	0.006 ± 0.003	0.006 ± 0.002
**Primary BAs**		0.06 ± 0.03	0.06 ± 0.03	0.03 ± 0.03	0.03 ± 0.02	0.009 ± 0.006	0.007 ± 0.004	0.006 ± 0.003	0.006 ± 0.002
**Secondary BAs**		0.09 ± 0.04	0.1 ± 0.04	0.08 ± 0.03	0.08 ± 0.01	0.001 ± 0.001	0.0008 ± 0.0005	0.0009 ± 0.0003	0.0008 ± 0.0003
**Total BAs**		0.14 ± 0.06	0.15 ± 0.06	0.11 ± 0.06	0.11 ± 0.03	0.009 ± 0.006	0.007 ± 0.005	0.007 ± 0.004	0.007 ± 0.002
**CBD (μg/mL)**		N.D.[a]	0.004 ± 0.002[b]	N.D.[a]	0.004 ± 0.004[b]	N.D.	N.D.	N.D.	N.D.

Targeted LC-MS analysis of BAs in colon content and liver from sham surgery (SS) and ovariectomized (OVX) mice treated with vehicle (VEH) or cannabidiol (CBD, 25 mg/kg body wt.). Each sample was injected in duplicate using negative and positive ionization. To prevent residual BAs from eluting into the subsequent sample, a twenty-minute column wash and calibration was performed between each sample injection. Individual BA concentrations were determined via external standard curves with internal standard calibration. Concentrations are reported as mean ± S.D. BAs not detected (N.D.) were assigned value of 0 for statistics. Differences between groups was assessed using 2-way ANOVA followed by the Benjamini Hochberg post-hoc test to correct for false-discovery rate (FDR). Different letters denote significant difference (q <0.05) when

comparing all groups. BA concentrations without any letters or asterisks were not significantly different between groups. BAs conjugated with either Taurine (T) or Glycine (G) were grouped as conjugated BAs, taurine, or glycine while unconjugated BAs refer to BAs that do not contain T or G in their structure. Primary BAs (PBA) refer to BAs that are produced by the host in the liver, and secondary BAs refer to PBAs that are microbially modified.
